# The association among thriving in life, quality of life, and suicidal ideation in Chinese urban older adults: the moderating effects of attitude toward own aging

**DOI:** 10.1186/s40359-024-01822-6

**Published:** 2024-05-30

**Authors:** Shu-e Zhang, Jiang-heng Liu, Yan-ping Wang, Qun-hong Wu, Zhong Zhang, Tao Sun, De-pin Cao

**Affiliations:** 1 Department of Health Management, School of Health Management, Harbin Medical University, Harbin, 150081 China; 2 Department of Health Policy and Management, School of Public Health, Hang Zhou Normal University, Hangzhou, 311121 China

**Keywords:** Thriving in life, Quality of life, Attitude toward own aging, Suicidal ideation

## Abstract

**Background:**

As the global trend of population aging intensifies, the health and well-being of the older population has gradually become a focus of attention for the global community. This study assessed the status of thriving in life among Chinese urban older adults and identified its relationship with attitude toward own aging and quality of life (QoL). It also tested whether attitude toward own aging moderates the association between thriving in life and Qol or between thriving in life and suicidal ideation.

**Methods:**

Primary data were collected through a cross-sectional survey among urban older adults from three provinces in China. They were invited to complete an anonymous survey using face-to-face interviews from December 2019 to January 2020. Data from 764 older adults were analyzed.

**Results:**

Approximately 44.39% of participants reported positive responses toward the four domains of thriving in life. Thriving in life and attitude toward own aging had a significant association with QoL. Thriving in life was a protective factor for suicidal ideation for older adults. Moreover, attitude toward own aging moderated the association between thriving in life and QoL and that between thriving in life and suicidal ideation.

**Conclusions:**

Chinese urban older adults were reportedly thriving in life, which contributed to increased QoL and reduced suicidal ideation. Notably, the study revealed that more positive attitudes towards own aging were associated with higher levels of thriving in life, better QoL, and reduced suicidal ideation. Targeted interventions for older adults should be devised to promote thriving in life and prevent negative attitudes of older people towards their own aging, further raising QoL and reducing suicidal ideation.

**Supplementary Information:**

The online version contains supplementary material available at 10.1186/s40359-024-01822-6.

## Background

The aging population is a global issue [1] that can be considered as one of the largest “Gray rhinos” for human society. “Gray rhinos” refer to high probability, huge impact, yet neglected threat. Governments globally face significant challenges and pressure in addressing the issue of aging, while healthcare systems of countries remain underprepared capacity for the growing aging population [2]. In China, the seventh population census conducted by the China National Bureau of Statistics in 2020 revealed important statistics. It showed that the number of individuals aged 60 and older reached 264 million, accounting for 18.70% of the total population. Additionally, the number of individuals aged 65 and older reached 191 million, accounting for 13.50% of the total population. China’s aging population is particularly characterized by a super-scale, ultra-fast speed, ultra-early stage, and ultra-stable structure. As a result, governments at all levels face increasingly significant challenges.

Today’s developing countries must adapt much more quickly to aging populations. In the industrialization and agricultural eras, disabled elderly people were unable to work and live independently. However, in the current digital age, older adults experience a new period of almost 10 years of healthy aging called the “third life period” that is characterized by living independently and working actively by older adults themselves. The third life period primarily refers to individuals over 60 years of age who are still able to work and live independently until they start to experience declines in their abilities. According to the latest census in China, this phenomenon is not limited to older people of a certain age but is inclusive of individuals who fall within the 60 to 80 age bracket. Additionally, advancements in healthcare have made it easier for people to live up to 80 years and beyond. In long-lived societies, the issues of quality of life and suicidal ideation of older adults are of great significance to their well-being. Improving the quality of life of older adults contributes to a healthy and productive old age, while reducing suicidal ideation contributes to the maintenance of their mental health. A healthy and happy older population is more likely to contribute meaningfully to society. To achieve positive and healthy aging, it is imperative for the government, society, and market to concentrate efforts on harnessing the potential strengths of these “long-life societies” and incorporating this societal transformation into strategies for healthy aging.

In this rapidly aging society, ensuring the well-being and continued contributions of older adult is crucial. This goal aligns with the theoretical frameworks of positive aging strategies and healthy aging strategies [3]. Positive psychology might offer a new perspective on cultivating a healthy and long-lived society for aging. It can guide interdisciplinary efforts and enhance our understanding of the functional mechanisms that promote well-being in older individuals. Further, positive psychology can shed light on the impact of different interventions on aging societies and assist policymakers in proposing practical aging-related policies and programs [4]. Currently, the predominant models for aging characterize it as a time of decline in functional abilities and independence. However, these models may not fully explain the positive social phenomena observed in older adults in contemporary society [5]. In a long-lived society, older adults not only need to live longer and healthier but also more meaningfully and happier. Contrasting these models, positive psychology characterizes old age as a time of well-being, even amid the presence of a mental disorder, and underlines the relevance of the concept of thriving in life. This has increased the interest of stakeholders in understanding the importance of positive experiences for older adults.

### Why explore the construct of thriving in life?

Thriving in life has been shown to be a key index for assessing the psychological growth of older adults. Nevertheless, the reality is that numerous studies having explored the concept of thriving among employees, students, and patients, with fewer having done so among older adults [6–8]. For example, in the higher education setting, thriving has been defined as being fully engaged intellectually, socially, and emotionally [8]. Meanwhile, thriving at work is described as a positive psychological state characterized by learning and vitality. This description aligns with prior psychological research, which emphasizes the importance of considering the affective and cognitive foundations of human growth [7]. Moreover, Schreiner described the thriving quotient in work-project [6], college [8], community [9], and classroom settings [10]. Furthermore, research has confirmed this thriving quotient was an effective evaluation indicator of students’ success [11]. Regarding the assessment of thriving among older adults, several instruments, such as the Thriving in Older Adult Life Assessment Scale, have been designed and applied in various cultures to assess thriving among older adults. The scale is available in multiple languages, including Chinese, Norwegian, and Swedish, and is considered reliable and valid for measuring thriving in long-term care facilities [12]. However, instruments such as this one were either devised for older people undergoing long-term care or with samples from Western countries. Hence, instruments for assessing thriving in life in general for older people in rapidly aging Eastern countries are urgently needed. Notably, over 80% of Chinese older adults receive gerontological care in their homes, highlighting significant differences between China and Western countries [11, 13]. Consequently, the previous instruments mentioned may not be suitable for comprehending thriving in life among Chinese urban older adults.

Based on research of thriving in long-term care and on our specific interest in aging societies with a Confucian culture, an assessment tool for thriving in life among urban Chinese older adults should include the constructs of vitality, learning, meaning, and pursuit. Learning refers to the feeling that older adults are adapting to the development of modern society through the acquisition and application of knowledge and skills. Vitality refers to the feeling of being full of energy and enthusiasm in the life of the older adults. Meaning refers to the degree of intimacy, harmony and coordination between the older adults and others in the process of life interactions or activities, reflecting the psychological state of the older adults seeking to meet their social needs. Pursuit refers to the mental state of the older adults to recognize and pursue the goal of their own life, and to strive to establish or increase the understanding of the connotation and goal of life in the present time, and to continuously maintain the ability of life activities and survival and development. Accordingly, we developed a research project aimed at developing a new instrument for assessing thriving in life among older adults in China that is suitable for the characteristics of the Chinese Confucian culture.

### Is thriving in life related to quality of life and suicidal ideation?

Quality of life has been shown to be a very useful indicator of overall health. According to the self-determination theory model by Ryan and Deci, higher thriving in life could increase optimal psychological functioning, further leading to higher QoL. According to the theory of flourishing, individual enthusiasm for life, productivity, engagement with others and in society, and resilience in the face of personal challenges contribute to physical and psychological well-being [14]. Thus, it may be that thriving in life is a protective factor of QoL.

Research shows that suicide is a leading cause of death worldwide and that suicide risk increases with age [15]. Despite the plethora of research on suicidal ideation, there is no consensus on its definition. One study defined suicidal ideation as a broad term used to describe a range of contemplations/wishes/preoccupations related to death/suicide [16]. Moreover, some studies include the construct of suicide planning within the notion of suicidal ideation, while others do not [17]. Research has shown that suicide prevention for older adults is challenging due to fewer warning signs and the greater use of deadlier methods [16]. The interpersonal theory of suicide proposed by Van Orden et al [18]. assumes that suicidal desire is determined by the co-existence of connectedness and perceived burdensomeness. Meanwhile, a study shows that older adults who possess a positive relationship and harmonious connection with others [19] had reduced suicidal ideation; that is, older adults who are thriving in life may generate greater constraints to engage in suicidal ideation. Therefore, we constructed two hypotheses: thriving in life has a positive association with the QoL of Chinese urban older adults (Hypothesis 1); and thriving in life has a negative association with suicidal ideation in Chinese urban older adults (Hypothesis 2).

### The relationship between thriving in life, attitude toward one’s own aging and QoL, and suicidal ideation

Socioemotional selectivity theory describes and explains the underlying mechanisms of age-related changes in social behavior [20]. The theory suggests that as individuals age, they become more focused on emotional goals and emotional well-being. This means that urban older adults may be more attentive to aspects related to their emotional and psychological well-being, and that urban older adults’ attitudes toward their own aging may influence their perceptions of and responses to these aspects. This is supported by research in positive psychology. Holding positive attitudes toward aging may lead older adults to be more optimistic and contented, thus enhancing their positive feelings and evaluations of life. Holding negative attitudes toward aging, on the other hand, may lead to negative emotional and psychological states, which in turn may affect life’s thriving and overall quality of life. Existing evidence shows that attitude toward own aging is associated with several detrimental psychological and physical outcomes among older adults [21]. Specifically, older adults with a more negative attitude toward own aging tend to encounter higher risks of negative psychological outcomes [22]. Attitude toward own aging has been defined as the combination of stable, integrative judgments that summarize the thoughts/feelings/memories of older people regarding their own aging or aging-related situations [23]. In older adults, attitude toward own aging was shown to be the strongest predictor of health-promoting behaviors [24, 25]; health-promoting behaviors, in turn, are crucial for older adults to remain functional, independent, and experience higher QoL. Research has also explored the mediating role of attitude toward own aging in the association between family environment and QoL [26], with a cross-national study showing that attitude toward own aging was the major mediator—followed by psychosocial loss and psychosocial growth—in the relationship between subjective health and QoL in older adults [27]. Attitude toward own aging was likely moderating the relationship between thriving in life and QoL in older adults, where a negative attitude buffered the strength of this relationship.

Nevertheless, the moderating effect of attitude toward own aging on this relationship has yet to be comprehensively explored by scholars. If older adults’ attitude toward own aging can indeed strengthen the aforementioned correlations, it may provide an important clue about how to improve QoL among Chinese urban older adults and which attitude toward own aging could be more beneficial to promote the positive effect of thriving in life. Therefore, we constructed two hypotheses: attitude toward own aging moderates the association between thriving in life and QoL (Hypothesis 3); and attitude toward own aging moderates the association between thriving in life and suicidal ideation (Hypothesis 4).

### Aim

Since China has been facing numerous challenges related to its rapidly aging population, strategies to assist the promotion/maintenance of older adults’ health and well-being have become vital. Thus, this study aimed to identify (1) the current status of thriving in life; (2) the association between thriving in life, QoL, and suicidal ideation; and (3) the moderating effect of attitude toward own aging in the relationship between thriving in life and QoL and between thriving in life and suicidal ideation among Chinese urban older adults.

## Methods

### Sample and data collection

The study was conducted according to the guidelines of the Declaration of Helsinki and approved by the Ethics Committee. The study used scientific and recognized measurement tools to evaluate variables such as the thriving in life, suicidal intention. For the suicidal intention survey, we again obtained the respondent’s consent and stated that it might produce discomfort in the test of suicidal ideation. Questionnaires were collected through face-to-face way, and trained investigators observed and paid much attention to the negative emotions of the participants throughout investigation process. In accordance with ethical requirements, the investigation can be terminated in the event of uncooperation and negative emotions. Furthermore, we employed a multi-stage stratified and convenience sampling method to collect primary data through a cross-sectional survey conducted in three provinces of China. The regions randomly sampled included Zhejiang province in the southeast and Heilongjiang province in the northeast. Sampling method took into account the economic and cultural characteristics of the numerous communities. The sample size calculation was based on a formula for estimating sample size in quantitative studies. Formula: $N=4{{\mu }_{\alpha }}^{2}{S}^{2}/{\delta }^{2}$, $\alpha$ is the confidence level, ${\mu }_{\alpha }$ represents the value of $\mu$ at confidence level $\alpha$, $S$is the standard deviation, and $\delta$ represents the standard error. In this study, a two-sided test was taken ($\alpha$=0.05, ${\mu }_{\alpha }$=1.96), and the tolerance error [0.25*S.*0.5*S*] was considered to be a reasonable range, which was calculated to give a sample size range of about 62 to 246 participants. Considering the required statistical indicators for questionnaire response rate, validity, and reliability during the tool development process, the minimum sample size was determined as 380 participants. Specifically, researchers were recruited in the early stages and systematic training was provided to the recruited investigators. Subsequently, fieldwork was conducted in which trained enumerators randomly selected different neighborhoods and surveyed older persons encountered at different times of the day. Finally, we recruited 764 older adults who yielded valid participants (effective response rate: 81.28%); they completed questionnaires via anonymous face-to-face interviews from October 2019 to December 2020. The eligibility criteria were being aged 60 years or older, residing in the city for more than six months, having no hearing or communication impairments, having no cognitive impairments, and volunteering to participate in the research.

### Measurement tools

#### Thriving in life

The thriving-in-life scale we used in this research was developed by us in a Chinese context [28]. The dimensions, sub-dimensions, and themes of the scale were identified through interviews with older adults, the thriving theory, and expert consultation, all of which provided the basic contents for us to develop the items and the structure of this measurement tool. Based on the collected data, we initially generated a pool of 56 items. These items were then subjected to multiple rounds of expert validation, resulting in a refined set of 28 items. To further refine the instrument, we applied exploratory factor analysis and confirmatory factor analysis, yielding 4 dimensions and 21 items questionnaire. Responses were provided on a five-point Likert scale, ranging from 1 to 5 (strongly inconsistent–strongly consistent). A sample item is “I feel alive and vital in daily life,” with total scores ranging from 21 to 105 and higher scores reflecting higher thriving in life. The Cronbach’s alpha was 0.932.

#### Attitude toward own aging

The attitude toward one’s own aging construct was measured using a five-item questionnaire derived from the short version of the Attitudes to Aging Questionnaire [29], which has been widely used in the Chinese context [30, 31]. Responses were provided on a five-point Likert scale, ranging from 1 to 5 (completely disagree–completely agree). A sample item is “I think I am old,” and higher scores reflected a more negative attitude toward own aging. In this study, the Cronbach’s alpha was 0.735.

#### Health-related QoL

To ensure that our survey was cost-effective and accessible, we used the eight-item short version (SF-8) to evaluate QoL [32]. It includes eight ordinal items: general health (GH), physical functioning (PF), physical roles (RP), bodily pain (BP), vitality (VT), social functioning (SF), mental health (MH), and emotional roles (ER). The SF-8 has shown good reliability/validity [32]. Responses were provided on a five-point Likert scale, ranging from 1 to 5 (very poor–very good). A sample item is “How would you describe your overall health,” and higher scores reflected a lower QoL. In this study, the Cronbach’s alpha was 0.876.

#### Suicidal ideation

Suicidal ideation was measured using the Chinese version of a four-item scale developed by Nugent and Cummings [33]. Participants responded on a five-point Likert scale, ranging from 1 to 5 (never–all the time). A sample item is “I feel that my life is over and I may as well end it,” and higher scores reflected higher suicidal ideation. In this study, the Cronbach’s alpha was 0.885.

### Statistical analysis

#### Preliminary analyses

We randomly divided the whole data set (*n* = 764) into two groups: Sample 1 (*n* = 380) and Sample 2 (*n* = 384). We applied exploratory factor analysis to Sample 1. Promax rotation with Kaiser normalization was applied to eliminate the entries with factor loadings. Promax rotation considers the correlation among components, and any resultant components were expected to be related. Kaiser normalization is a widely used factor rotation method that makes the results of the analysis more interpretable and understandable [34]. For Sample 2, we conducted confirmatory factor analysis in order to assess the validity of our thriving-in-life scale. We used Pearson’s correlation coefficients to estimate correlations among thriving in life, QoL, and suicidal ideation. We set statistical significance at a *P* < 0.05, and all aforementioned analyses were conducted using SPSS 24.0 and Amos 24.0 for Windows.

#### Moderator analysis

We conducted hierarchical linear regression analysis to test the effects of thriving in life on QoL and on suicidal ideation. The analytical data were described by *F*, *R*^*2*^, and *R*^*2*^-changes, and we assessed the fit of the model using *R*^*2*^. We obtained standardization regression coefficients (*β*) and *P* values for each step in the regression model. Upon finding an interaction effect that was statistically significant, we conducted a simple slope analysis to visualize the interaction term.

## Results

### Participants’ demographics and thriving in life

In our sample, the mean age was 71.83 (standard deviation [SD] = 7.45, range: 60–96); 54.19% of the participants were female; about 80.1% were married, 1.2% were divorced, and 18.7% were widowed; 23.04% did not complete primary school, 35.73% completed primary school, 20.55% completed middle school, 12.96% completed high school, and 7.33% had higher education; approximately 44.37% received pensions; and about 76.70% defined their economic status as average and 10.99% as above average (Table 1).

**Table 1:** Participants’ demographic information

Characteristics	*N* (%)		Characteristics	*N* (%)	
	*N*	%		*N*	%
Sex			Education		
Male	346	45.29	No school	176	23.04
Female	414	54.19	Primary school	273	35.73
**Age**			Middle school	157	20.55
≤ 65	167	21.86	High school	99	12.96
66–70	217	28.40	Higher education	56	7.33
71–75	161	21.07	Unsure	3	0.39
76–80	115	15.05	**Marital status**		
≥ 81	104	13.61	Married	611	80.10
**Pension**			Divorce	9	1.20
Yes	339	44.37	Widowed	143	18.70
No	384	50.26	**Monthly income - Monthly expenditure**		
Unsure	41	5.37	Below zero	21	2.75
**Register**			Zero	73	9.55
Migrate to City	437	57.20	Above zero	586	76.70
Urban-local	326	42.67	Unsure	84	10.99

### Exploratory factor and reliability analyses of the thriving-in-life scale

Our exploratory factor analyses revealed a Bartlett spherical test coefficient was less than 0.001, representing an obvious level of significance. The Kaiser-Meyer-Olkin coefficient was 0.919, which was greater than 0.900. The Bartlett spherical test illustrated that there were several common factors among the 28 items.

Initially, the principal component analysis showed that two items needed deletion owing to having four mutual factors; ultimately, it suggested a four-factor structure, accounting for 61.095% of the variances (Factor 1: 17.117%; Factor 2: 15.169%; Factor 3: 14.507%; and Factor 4: 14.302%). The Cronbach’s α for the dimensions of the Life Thriving Measurement Instrument were 0.786, 0.820, 0.857, and 0.860, respectively. The scale showed an overall Cronbach’s α of 0.932, indicating high internal consistency. Moreover, the standardized factor loading of all the item ranges was above the threshold limit of 0.5—as suggested by Hair et al. [35] in their research. Furthermore, there was all statistically significant positive correlations between fours sub-factors of the thriving-in-life scale (*P* < 0.01). Thus, all items showed good internal consistency for the analyses in Sample 1 (*n* = 384; Table 2).

**Table 2:** Item loading and sub-factors’ inter-correlations of the thriving-in-life scale in Sample 1 (*n* = 384)

Sub-factors	Items	F1	F2	F3	F4
F4	IT1	-0.031	0.080	-0.068	**0.887**
	IT2	-0.019	0.076	-0.017	**0.832**
	IT3	0.120	0.050	0.120	**0.605**
	IT4	-0.013	-0.286	-0.023	**0.536**
	IT6	-0.015	-0.158	-0.136	**0.519**
	*Inter-correlation (r)*	*0.502* ^**^	*0.583* ^**^	*0.597* ^**^	*1.000*
F3	IT7	-0.003	-0.311	**-0.541**	0.151
	IT8	-0.087	-0.443	**-0.544**	0.099
	IT9	0.135	0.256	**-0.793**	-0.018
	IT10	0.002	-0.191	**-0.632**	0.056
	IT11	0.300	0.003	**-0.618**	-0.002
	*Inter-correlation (r)*	*0.633* ^**^	*0.693* ^**^	*1.000*	*0.597* ^**^
F2	IT12	0.092	**-0.526**	-0.237	0.129
	IT13	0.141	**-0.599**	-0.108	0.040
	IT15	0.167	**-0.599**	0.053	0.151
	IT16	0.075	**-0.767**	0.097	-0.057
	IT18	0.247	**-0.561**	-0.136	0.024
	*Inter-correlation (r)*	*0.685* ^**^	*1.000*	*0.693* ^**^	*0.583* ^**^
F1	IT21	**0.760**	-0.068	0.112	0.112
	IT22	**0.796**	0.022	0.007	0.086
	IT23	**0.480**	-0.166	-0.089	0.048
	IT24	**0.815**	0.008	-0.059	-0.084
	IT25	**0.814**	-0.025	-0.088	-0.070
	IT26	**0.662**	-0.064	-0.050	0.043
	*Inter-correlation (r)*	*1.000*	*0.685* ^**^	*0.633* ^**^	*0.502* ^**^

Note: F1: perceived harmonious relations; F2: live for the moment; F3: maintain personal growth; F4: Maintain vitality; IT: item. ^**^P < 0.01

### Confirmatory factor analysis of the thriving-in-life scale

**Fig. 1:**
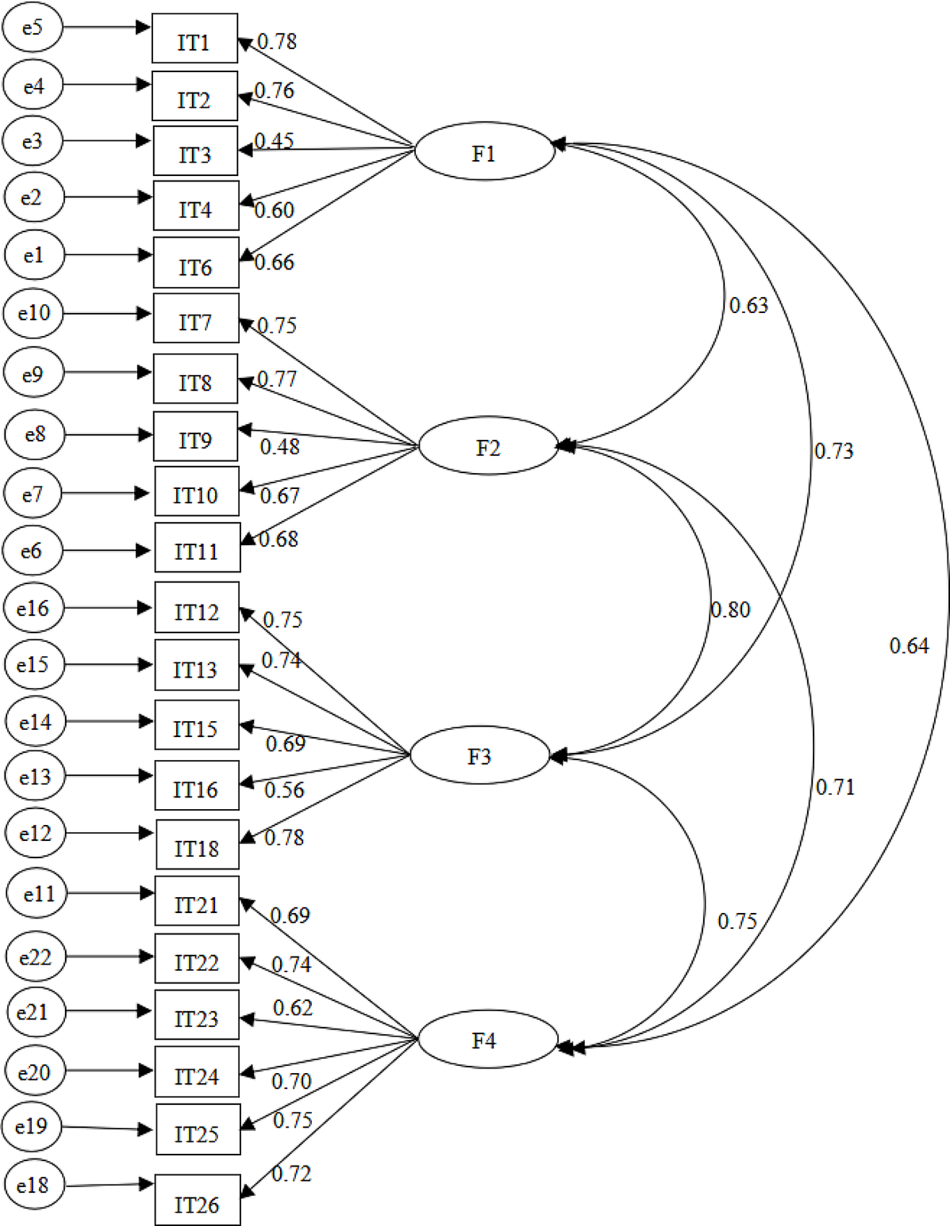
Standardized 4-factor structural equation model

To evaluate the goodness of the fit indexes of the models, including χ^2^/df (≤ 3) [36], the goodness of fit index, comparative fit index, and Tucker-Lewis index, we used the following cut-off values: <0.90 as a lack of fit, 0.90–0.95 as a good fit, and > 0.95 as excellent fit [37]. Finally, we used the root mean square error of approximation, with values < 0.05 showing an excellent fit and those between 0.05 and 0.08 showing an acceptable fit. In our four-factor model, all indexes showed appropriate goodness of fit to the data (Fig. 1; Table 3) [38].

**Table 3:** Tests of measurement invariance for the four-factor model in Sample 2 (*n* = 380)

χ2	df	*p*	χ2/df	CFI	GFI	RMSEA	SRMR
426.788	181	0.000	2.358	0.930	0.903	0.060	0.0372

Note: df: degree of freedom; CFI: comparative fit index; GFI: goodness of fit index; RMSEA: root mean square error of approximation; SRMR: standardized root mean square residual

Figure 1 Confirmatory bifactor model representing thriving in life (TIL), and its four factors of perceived harmonious relations (F1), live for the moment (F2), maintain personal growth (F3), maintain vitality (F4) based on the 21-item thriving in life scale. See Table 3 for item loadings and the index for all scale items in Sample 2 (*n* = 380).

### Correlations among variables of interest

**Fig. 2:**
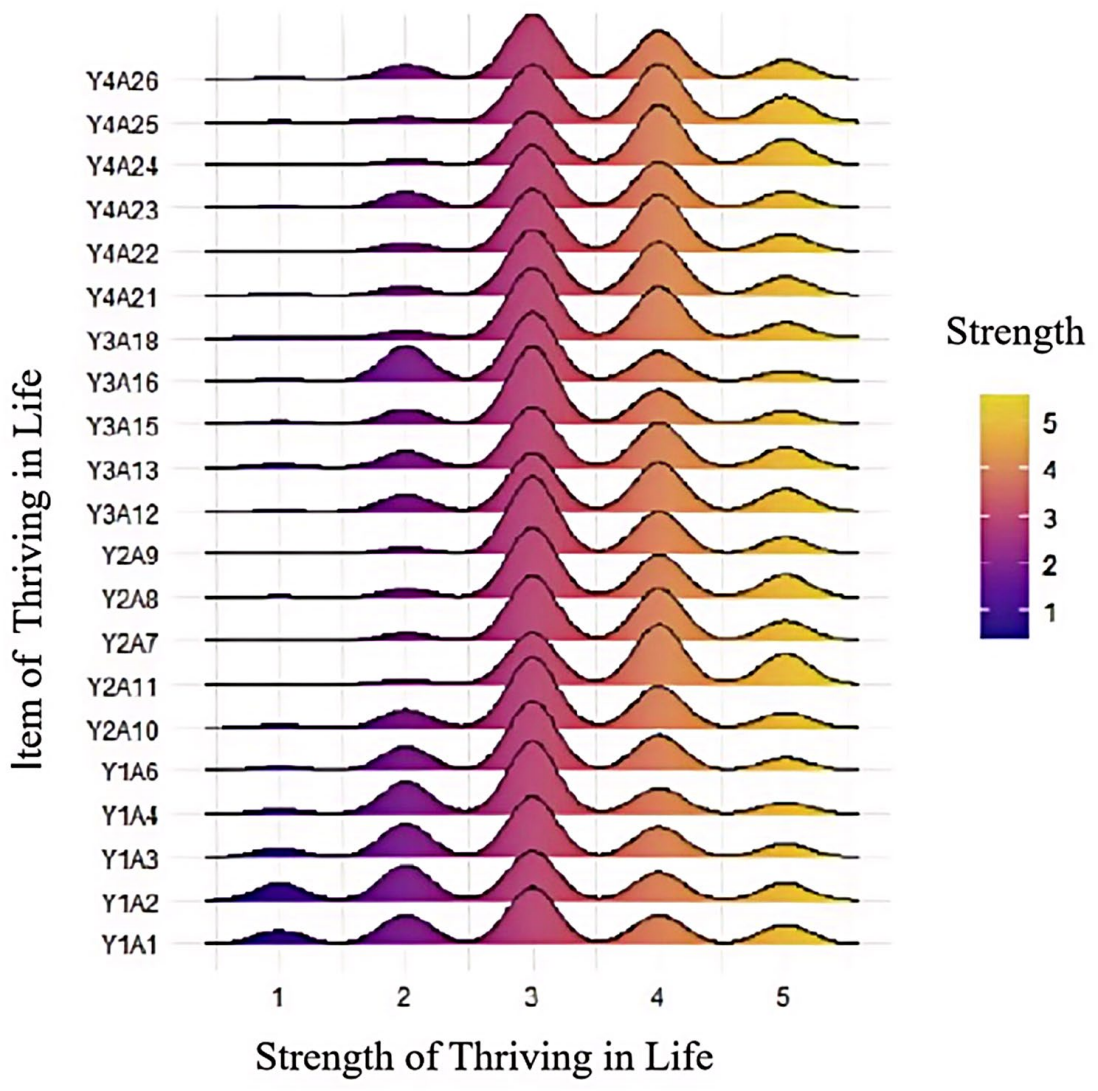
Ridge map of the thriving degree proportion of 21 projects among older adults

To assess thriving in life, we considered the responses “strongly consistent” or “consistent” as positive responses, and all others as negative responses (Fig. 2; Table 4). In our total sample (*n* = 764), approximately 44.39% provided positive responses; from highest to lowest, they provided the most positive responses to the following topics: perceived harmonious relations (61.02%), maintain personal growth (47.09%), live for the moment (39.17%), and maintain vitality (30.29%).

**Table 4:** Means, standard deviation, and positive response of participants to thriving in life (*n* = 764)

Variables	M	SD	Positive responses (%)	Rank
Maintain vitality	3.06	0.756	30.29%	4
Maintain personal growth	3.53	0.636	47.09%	2
Live for the moment	3.38	0.659	39.17%	3
Perceived harmonious relations	3.57	0.637	61.02%	1
Thriving in life	3.39	0.554	44.39%	—

Note: SD: standard deviation

Using Pearson’s correlation coefficients, we observed that all continuous variables of interest were significantly correlated with each other. Thriving in life was positively correlated with QoL (*r* = 0.489, *P* < 0.01), as well as negatively correlated with negative attitude toward own aging (*r*=-0.491, *P* < 0.01) and suicidal ideation (*r*=-0.250, *P* < 0.01). Moreover, negative attitude toward own aging was negatively correlated with QoL (*r*=-0.430, *P* < 0.01) and positively correlated with suicidal ideation (*r* = 0.209, *P* < 0.01).

### Hierarchical regression analyses

**Fig. 3:**
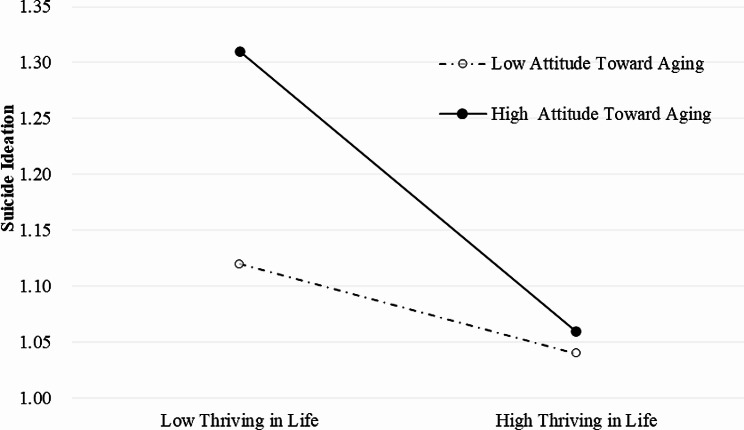
Simple slope plot of the interaction between thriving in life and attitude toward own aging and the impact on suicidal ideation

Hierarchical linear regression analysis was performed to test the effects of thriving in life on QoL. In the first step, we examined the influence of the control variables, including age, marital status, older age group, retirement, and education. In the second step, thriving in life was found to be significantly and positively related to QoL (*β* = 0.360, *P* < 0.01), while attitude toward own aging was significantly and negatively associated with QoL (*β*=-0.208, *P* < 0.01). Thriving in life and attitude toward own aging improved the model fit for QoL (*adjusted R*^*2*^ = 0.303, *R*^*2*^ = 0.211, *P* < 0.01). The interaction term of thriving in life and attitude toward one’s own aging was significantly and negatively associated with QoL (*β* = 0.640, *P* < 0.01). The impact of thriving in life on QoL was different in low (1 SD below the mean, *P* < 0.001) and high (1 SD above the mean, *P* < 0.001) levels of attitude toward own aging (interactions are shown in Fig. 3). Then, a simple slope analysis revealed that when attitude toward own aging is higher, the strength of association between thriving in life and QoL becomes stronger.

**Fig. 4:**
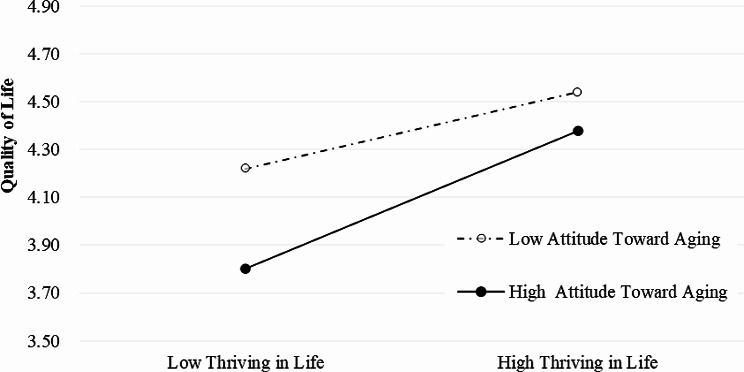
Simple slope plot of the interaction between thriving in life and attitude toward own aging and the impact on quality of life

Moreover, thriving in life was found to be significantly and negatively associated with suicidal ideation (*β*=-0.217, *P* < 0.01), and thriving in life and attitude toward own aging improved the model fit for suicidal ideation (*adjusted R*^*2*^ = 0.089, *R*^*2*^ = 0.070, *P* < 0.01). Further, the interaction term between thriving in life and attitude toward own aging was significantly and negatively associated with suicidal ideation (*β*=-0.588, *P* < 0.01). The impact of thriving in life on suicidal ideation was different in low (1 SD below the mean, *P* < 0.001) and high (1 SD above the mean, *P* < 0.001) levels of attitude toward own aging (interaction is shown in Fig. 4; Table 5). A simple slope analysis revealed that when attitude toward own aging is higher, the strength of association between thriving in life and suicidal ideation becomes stronger.

**Table 5:** Results of the hierarchical linear regression models (*n* = 764)

Variables	Quality of life		Suicidal ideation	
	*M*_*1*_ *(β)*	*M*_*2*_ *(β)*	*M*_*3*_ *(β)*	*M*_*4*_ *(β)*
**Control variables**				
Sex	0.072^*^	0.069^*^	-0.042	-0.045
Age	0.122^**^	0.128^**^	0.081^*^	0.083^*^
Marital status	0.070^*^	0.061^*^	0.003	-0.005
Migrating older	-0.020	-0.023	0.062	0.060
Retirement	-0.008	-0.005	0.096	0.098^*^
Education	0.035	0.030	0.029	0.024
**Independent variable**				
Thriving in life	0.360^**^	0.202^**^	-0.217^**^	0.296
**Moderator Variable**				
Attitude toward own aging	-0.208^**^	-0.891^**^	0.072	0.693^**^
**Interaction**				
Thriving in life and attitude toward own aging		-0.643^**^		-0.588^**^
*F*	39.502^**^	8.797^*^	8.719^**^	8.976^**^
*R* ^*2*^	0.303^**^	0.309^**^	0.087^**^	0.089^**^
*∆R* ^*2*^	0.211^**^	0.015^**^	0.057^**^	0.070^**^

Note: *P < 0.05; **P < 0.01. M1 and M3 are models that include control variables and do not include interaction terms

## Discussion

### The thriving-in-life scale

Spreitzer et al. [39] conceptualized thriving into two dimensions in a work context: vitality and learning. Moreover, thriving is closely linked to the physical or living environment and has been explored in relation to infancy, adolescence, and older people living in nursing homes [40]. However, we determined that some minor changes were found in regard to its construct from the thriving-in-life scale in the current study. The exploratory factor analysis demonstrated that thriving-in-life scale in the current study (account variance 61.095%) should have a four-factor structure, with each factor describing a differential aspect of thriving: perceived harmonious relations, live for the moment, maintain personal growth, and maintain vitality. Further, our results showed that the scale had appropriate construct validity and was well supported by the confirmatory factor analysis models. Differences in the Chinese version of the Thriving of Older People Assessment Scale in long-term care and the results indicate that the thriving-in-life scale was a measure of thriving in life among older urban Chinese people [7].

Furthermore, our results showed that the mean score of thriving in life of Chinese urban older adults in our sample was 3.386 ± 0.554, which was higher than the median score. According to the 47th Chinese Statistical Report on Internet Development by the China Internet Network Information Center [41], the number of netizens aged 60 years and older has doubled since the beginning of the COVID-19 outbreak, having reached 11.8 million [42]. Currently, individuals over 60 years of age are generally able to maintain their ability to work and live independently, and compared with older adults from prior decades, Chinese urban older adults are no longer just “forced” to survive; instead, they tend to currently live longer and happier lives, having a generally higher QoL in the digital era. This allows for the rationale that, in the current digital society, older people are productive and valuable for society. Therefore, stakeholders should endeavor to make adequate use of older human capital, an intervention that can help us construct a positive aging society. Furthermore, it is an investment in the well-being and intrinsic capacity of older people as productive and valued members of society. Therefore, this study provides basic data for stakeholders to develop strategies aimed at increasing the thriving in life among Chinese urban older adults, which should include policies, construction of community environments favorable to such thriving, and educational activities on the topic.

### Urban older adults with a higher thriving in life have a higher QoL and lower suicidal ideation

Our findings show that thriving in life was positively associated with QoL and negatively associated with suicidal ideation among Chinese urban older adults. Urban older adults with a higher thriving in life can manage the interpersonal relationships with themselves and others to obtain more social support and positive affect, which can further improve their physical, social, and mental health, thus leading to a higher level of QoL and lower level of suicidal ideation. Indirect support for the effects of thriving in life on QoL and suicidal ideation comes from Maslow’s theory of Hierarchy of Needs, indicating that living conditions with thriving for competency, autonomy, and relatedness are associated with greater QoL and lower suicidal ideation among older adults [43]. Thriving is related to a composite state that exhibits the following aspects: making the best of the situation, taking part in activities, and maintaining social relationships by the capacity and wishes. Older adults who are thriving in life tend to pursue goals that are important, fulfilling, challenging, and fueled by curiosity about the world. Previous research has proposed that the older adults’ social networks affected both physical and psychological health through a combined effect of buffering negative emotions and promoting health relevant behaviors [43]. Therefore, older adults who are thriving in life engage more easily in social participation, have confidence in life, obtain more social support, resulting in further preventive health behaviors [44]. These factors may promote psychological well-being for elderly people, evoke positive affect, and allow these older adults to live longer than their counterparts who are not thriving in life. In addition, thriving older adults contribute to objective psychological prosperity and social connectedness, which can promote an increase in their QoL, with a decline in suicidal tendencies caused by dilemmas such as disability, infirmity, social isolation, and loneliness.

### A negative attitude toward own aging strengthens the impact of thriving in life on QoL

Our findings show that thriving in life was positively associated with QoL among Chinese urban older adults, while attitude toward own aging was negatively associated with their QoL. Results of the simple slope analysis showed that the stronger effect of thriving in life on the QoL among older adults exists along with more negative attitudes toward own aging. One study demonstrated that a positive attitude toward own aging helps elderly people reduce negative psychological symptoms, promote healthy behavior, and that older adults with such a positive attitude had higher QoL [43]. Moreover, in older adults, attitude toward own aging has been linked to several detrimental psychological/physical outcomes [44], including anxiety, depression [21], and more cardiovascular stress and mental illness [45]. Our evidence also depicts the moderating effect of attitude toward own aging on the association between thriving in life and QoL, where this association was stronger among older people with a more negative attitude toward own aging than among their counterparts. Furthermore, older adults with a positive attitude toward own aging may incur fewer negative emotions and effects in their life course, allowing for the influence of thriving in life on QoL to be increased [46]. In contrast, among older adults, having a negative attitude toward own aging was shown to be a risk factor for age-vulnerable cognitive abilities [47], while increasing the risk of experiencing mental illnesses and having a reduced will to live. These characteristics, in turn, are likely to reduce the potential impact of thriving in life on QoL. Knowledge about this moderating mechanism may allow for stakeholders to develop more effective strategies for preventing Chinese older adults from incurring negative attitudes toward their own aging and/or to foster more positive life attitudes, thereby promoting QoL. Our findings demonstrate that interventions about negative attitudes toward one’s own aging are needed for modification to increase the potential for older adults to experience well-being and achieve active aging. Relevant interventions such as providing opportunities for older adults to engage in meaningful activities that promote self-esteem and self-efficacy, enhancing social support and connection, and promoting positive aging messages and images in the media and society. In addition, community education programs, workshops, and counseling sessions can be implemented to challenge age-related stereotypes, provide accurate information about the aging process, and promote positive self-perceptions of aging in older adults.

### A negative attitude toward own aging strengthens the impact of thriving in life on suicidal ideation

Our results found that thriving in life was negatively associated with suicidal ideation while attitude toward own aging was not, and that attitude toward own aging moderated the association between thriving in life and suicidal ideation. Results of the simple slope analysis showed that the stronger effect of thriving in life on the suicidal ideation among older adults exists along with more negative attitudes toward own aging. Specifically, the higher the negative attitude toward own aging, the stronger the effect of thriving in life on suicidal ideation.

A negative attitude toward own aging involves a negative age stereotype that allows for older adults to negatively adjust their psychological expectations about their own aging experience and contributes to their lower levels of well-being and identification. Accordingly, Chinese urban older adults who have a more negative attitude toward own aging may be at a higher risk of detrimental outcomes related to physical and mental health. This is because this negative attitude may be capable of regulating their positive emotions, further constraining their ability to recognize and promote psychological prosperity. There is a plethora of data showing that negative attitude toward own aging in older adults predicts subsequent physical disabilities [48], cognitive impairment, hopelessness, loneliness, and increases the likelihood of experiencing lower help-seeking willingness and sense of connectedness [19], all which may induce the occurrence of suicidal ideation. Furthermore, with a more negative attitude toward own aging, older adults may lack of confidence in life, reaching a deadlock and becoming more vulnerable [19], having a weakened desire to live. Therefore, older adults with a negative attitude toward their own aging may have a weaker desire to be recognized and respected, and the influence of thriving in life on suicidal ideation is increased. Thus, we believe that the significant effect of thriving in life on suicidal ideation should be highlighted when policymakers formulate solutions for preventing/reducing suicidal ideation in Chinese urban older adults. Potential interventions include encourage older adults to actively participate in community activities, volunteer work. Engaging in meaningful activities and giving back to the community can enhance a sense of worth, social integration, and personal fulfillment, thereby promoting a thriving life. In addition, Governments should provide opportunities for individual development, lifelong learning and skills acquisition for older adults. This could include vocational training, recreational activities and educational programs that would enable older adults to pursue new interests, enhance their self-esteem and develop their sense of accomplishment. Besides, the government can provide a social support network for the older adults by creating senior community centers or senior clubs. These measures will reduce suicidal ideation among the older adults while promoting prosperity in their lives. Furthermore, although our findings showed that attitude toward own aging is not directly related to suicidal ideation, it may still be indirectly related, making it a potential target variable when devising interventions aimed at reducing suicidal ideation. Despite these contributions, we see space for future research to strengthen our findings by replicating our examinations within samples from different cultures.

### Limitations

This study explored thriving in life among older adults from the perspective of life sequence, which is a continuation of the research on thriving at work, thriving at learning, and thriving at infants, and further broadens the research scope of thriving. First, we used convenience sampling for participant recruitment, which may have resulted in a sampling bias and have made our sample non-representative of the entire Chinese population. Second, the older adults in our sample might have provided responses to conform to social norms/expectations, and there were no methodological efforts on our part to address this reality; thus, we cannot entirely rule out social desirability bias, which denotes that our results may not be completely accurate for the target population. Furthermore, we used a descriptive correlational study design, denoting that we cannot infer causal relationships; this makes longitudinal studies on the same variables warranted to ascertain their causal associations. Considering the huge differences between urban and rural elderly people in China, the current study only focused on thriving in life among Chinese urban older adults, which deserves further study.

## Conclusions

Our self-developed 21-item thriving-in-life scale was shown to have a four-factor structure, with each factor representing different aspects of thriving in life: perceived harmonious relations, live for the moment, maintain personal growth, and maintain vitality. Our preliminary analyses showed that the scale was reliable and valid for use with Chinese urban older adults. The current study provided a new perspective of how to foster a sense of well-being despite physical deterioration among older adults. Moreover, our evidence showed that Chinese urban older adults were generally thriving in life, and this contributed to increasing their QoL and reducing suicidal ideation. We also showed that attitude toward own aging moderated the association between thriving in life and QoL and on the association between thriving in life and suicidal ideation; specifically, the higher the negative attitude toward own aging, the higher the effect of thriving in life on QoL and on suicidal ideation. Thus, policymakers should develop targeted interventions aimed at modulating the constructs of thriving in life and attitude toward own aging in Chinese urban older adults, in turn improving QoL and reducing suicidal ideation in the target population.

### Electronic supplementary material

Below is the link to the electronic supplementary material.

Supplementary Material 1

## Data Availability

The datasets used and/or analysed during the current study are available from the corresponding author on reasonable request.

## Abbreviations

BP: Bodily Pain
ER: Emotional Roles
GH: General Health
MH: Mental Health
PF: Physical Functioning
QoL: Quality of Life
RP: Physical Roles
SF: Social Functioning
VT: Vitality
