# Supplementary material for: The association among thriving in life, quality of life, and suicidal ideation in Chinese urban older adults: the moderating effects of attitude toward own aging
Source: BMC Psychol. 2024 May 30;12:315. doi: 10.1186/s40359-024-01822-6 (PMC11140986; doi:10.1186/s40359-024-01822-6)
Supplement: Supplementary file 1 — Supplementary Material 1 [file 40359_2024_1822_MOESM1_ESM.docx]

**APPENDIX**

**Measurement Tool of Thriving in Life**

Measurement tool of thriving in life of the urban older adults. Scoring: A 5-point Likert scale was used, ranging from 1–5 (strongly inconsistent–strongly consistent), and the higher the score, the higher the thriving in life of the urban older adults.

| **Serial number** | **Items** | **Dimension** |
| --- | --- | --- |
| 1 | For the last two weeks, you have always maintained the habit of studying regularly. | Maintain personal growth  (Learning) |
| 2 | The saying goes, 'It is never too old to learn.' You are now continuously learning new skills. For example, learning to use a smartphone or engaging in activities such as dancing, painting, or playing chess." |  |
| 3 | As you get older, you change habits you think are bad or wrong (stereotypes). |  |
| 4 | As you get older, you accept some of the new perspectives, ideas and behaviors of today's youth. |  |
| 6 | At this stage, you are looking forward to your future life and want to know what's new in the society. |  |
| 7 | Now at this stage, you always do what makes you happy. | Live for the moment  (Meaning) |
| 8 | At this stage, if you encounter difficulties in life, you are able to think rationally and maintain a positive outlook. |  |
| 9 | At this stage, the future is uncertain, and you believe that making the most of today is more important. |  |
| 10 | At this stage, you are able to accept whatever may happen in the future with equanimity. |  |
| 11 | At this stage, you always prioritize happiness and joy. |  |
| 12 | At this stage, you always feel energized and full of vitality | Maintain vitality  (Vitality) |
| 13 | In the past two weeks, you have been living a fulfilling life with plenty of things to do. |  |
| 15 | In the past two weeks, you have felt that life has momentum and purpose. |  |
| 16 | In the past two weeks, you have consistently had a clear mind and focused attention. |  |
| 18 | At this stage, every day for you is meaningful and significant. |  |
| 21 | In the past two weeks, you have been needed by your family or friends. | Perceived harmonious relations  (Pursuit) |
| 22 | In the past two weeks, you have been important to your family and friends. |  |
| 23 | In the past two weeks, you have been consistently meeting with or talking to your close friends. |  |
| 24 | In the past two weeks, your family has been caring about your life. |  |
| 25 | In the past two weeks, the people around you (family or friends) have shown respect towards you. |  |
| 26 | In the past two weeks, you have desired to spend time with friends or family, staying in touch, discussing daily life, sharing happy or unhappy events. |  |
